# MXene-Coated Ion-Selective Electrode Sensors for Highly Stable and Selective Lithium Dynamics Monitoring

**DOI:** 10.1021/acs.est.3c06235

**Published:** 2023-12-11

**Authors:** Yuankai Huang, Moyosore A. Afolabi, Lan Gan, Su Liu, Yongsheng Chen

**Affiliations:** School of Civil and Environmental Engineering, Georgia Institute of Technology, Atlanta, Georgia 30332, United States

**Keywords:** water sensor, ion selective electrode, MXene composite membrane, sulfonate group, resource recovery, antifouling

## Abstract

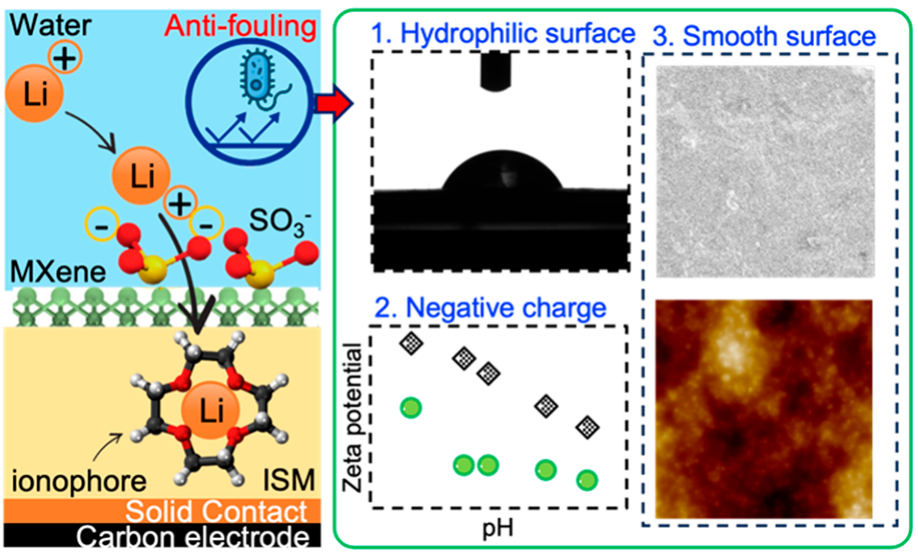

Lithium holds immense significance in propelling sustainable energy and environmental systems forward. However, existing sensors used for lithium monitoring encounter issues concerning their selectivity and long-term durability. Addressing these challenges is crucial to ensure accurate and reliable lithium measurements during the lithium recovery processes. In response to these concerns, this study proposes a novel approach involving the use of an MXene composite membrane with incorporated poly(sodium 4-styrenesulfonate) (PSS) as an antibiofouling layer on the Li^+^ ion selective electrode (ISE) sensors. The resulting MXene-PSS Li^+^ ISE sensor demonstrates exceptional electrochemical performance, showcasing a superior slope (59.42 mV/dec), lower detection limit (10^–7.2^ M), quicker response time (∼10 s), higher selectivity to Na^+^ (−2.37) and K^+^ (−2.54), and reduced impedance (106.9 kΩ) when compared to conventional Li^+^ ISE sensors. These improvements are attributed to the unique electronic conductivity and layered structure of the MXene-PSS nanosheet coating layer. In addition, the study exhibits the long-term accuracy and durability of the MXene-PSS Li^+^ ISE sensor by subjecting it to real wastewater testing for 14 days, resulting in sensor reading errors of less than 10% when compared to laboratory validation results. This research highlights the great potential of MXene nanosheet coatings in advancing sensor technology, particularly in challenging applications, such as detecting emerging contaminants and developing implantable biosensors. The findings offer promising prospects for future advancements in sensor technology, particularly in the context of sustainable energy and environmental monitoring.

## Abstract 2

This study introduces MXene-poly(sodium 4-styrenesulfonate) composite membrane as the antibiofouling layer on Li^+^ ISE sensors, enabling accurate, long-term lithium monitoring crucial for optimizing lithium recovery processes.

## Introduction

Lithium has become a crucial element for advancing sustainable energy and environmental systems, and the United States Geological Survey (USGS) designated lithium as a “critical” mineral for the U.S. economy and national security.^1^ Recycling techniques, such as membrane filtration^2,3^ have been developed for lithium recovery from various water streams like salt-lake brines (carry lithium in concentrations as high as 10^2^–10^3^ ppm) (Table S1). These techniques serve as an alternative to the traditional hard rock mining of ores,^4^ aiming to reduce reliance on international supply chains. However, to ensure the successful implementation of cost-effective and energy-efficient lithium recovery processes, it is imperative to accurately quantify lithium dynamics and develop multiobjective programmable models for system visualization and process control.^5^

The current primary methods for lithium monitoring, including atomic absorption spectroscopy^6^ and inductively coupled plasma mass spectrometry,^7^ are encumbered by the necessity for extensive sample preparation and are characterized by high operational costs and intricate procedural requirements, thus precluding the realization of real-time lithium monitoring throughout the recovery process. While fluorescence methods serve as viable candidates for real-time lithium monitoring, their accuracy in detection is highly susceptible to environmental variables such as pH and temperature.^8^ By contrast, electrochemical sensors, specifically potentiometric ion-selective electrode (ISE) sensors, have shown remarkable potential for real-time alkali metals monitoring due to their rapid response time, cost-effectiveness, and extensive detection range.^9,10^ However, using polyvinyl chloride (PVC) membrane-based ISE sensors for lithium monitoring presents challenges in selectivity^11^ and durability,^12^ both of which must be addressed to ensure precise and dependable lithium measurements in waste streams. The presence of interference ions in water and wastewater streams, specifically K^+^ (0.331 nm), Na^+^ (0.358 nm), and NH_4_^+^ (0.331 nm), exhibit similar hydrated ionic radii and valence comparable to Li^+^ (0.382 nm) and can pose challenges in selectivity (Table S1). These ions have the potential to influence the complexation behavior between themselves and the ionophore- or cavity-based selectors intrinsic to the ISE membrane. Their mere presence can shift the ion distribution equilibrium between the membrane and the analyte.^13^ The durability issue mainly comes from the biofilm accumulation on the membrane surface, obstructing the diffusion of lithium ions to the sensing layer and causing sensor reading drift over time.^14^ While antimicrobial materials, such as 6-chloroindole^15^ and silver nanoparticles,^16^ have demonstrated potential in inactivating bacteria, they fall short in preventing the initial adhesion of bacteria to the membrane surface. An alternative approach involves modifying the membrane surface morphology using antiadhesive materials like polydopamine (PDA)^17^ or poly(ethylene glycol) (PEG).^18^ Nonetheless, the employment of these materials presents new challenges, as they either obstruct the primary ion diffusion or promote the extraction of interfering ions from the sample solution, ultimately compromising the sensors′ selectivity.^19^

MXene is a two-dimensional (2D) material with abundant surface functional groups, such as hydroxyl (−OH), oxygen (−O), and fluorine (−F), that encourage hydration layers on the surface, acting as barriers particularly against bacterial foulants, effectively preventing bacterial adhesion on the sensor surface when exposed to wastewater.^20^ The tunability of MXenes enables control over interlayer spacing and surface chemistry, which permits the optimization of membrane properties, including fouling resistance, by tailoring surface properties and pore sizes to minimize foulant adhesion and infiltration.^21^ Additionally, the incorporation of functional additives, such as the sulfonate (SO_3_H) group, into the MXene matrix can contribute to improved Li^+^ selectivity.^22^ Our previous research has demonstrated that incorporating the SO_3_H group into the MXene matrix resulted in enhanced Li^+^/Na^+^ and Li^+^/K^+^ selectivity, as the binding energy between Li^+^ and the SO_3_H group (−0.220 eV) is lower than that of Na^+^ (−0.223 eV) and K^+^ (−0.229 eV) according to density functional theory (DFT) calculations.^23^ The exceptional electrical conductivity of MXenes can also enhance the signal strength, sensitivity, and response time of the ISE sensors.^24^ Overall, these properties make MXene a promising candidate for antifouling coatings in ISE sensor applications with high Li^+^ selectivity.

**Figure 1:**
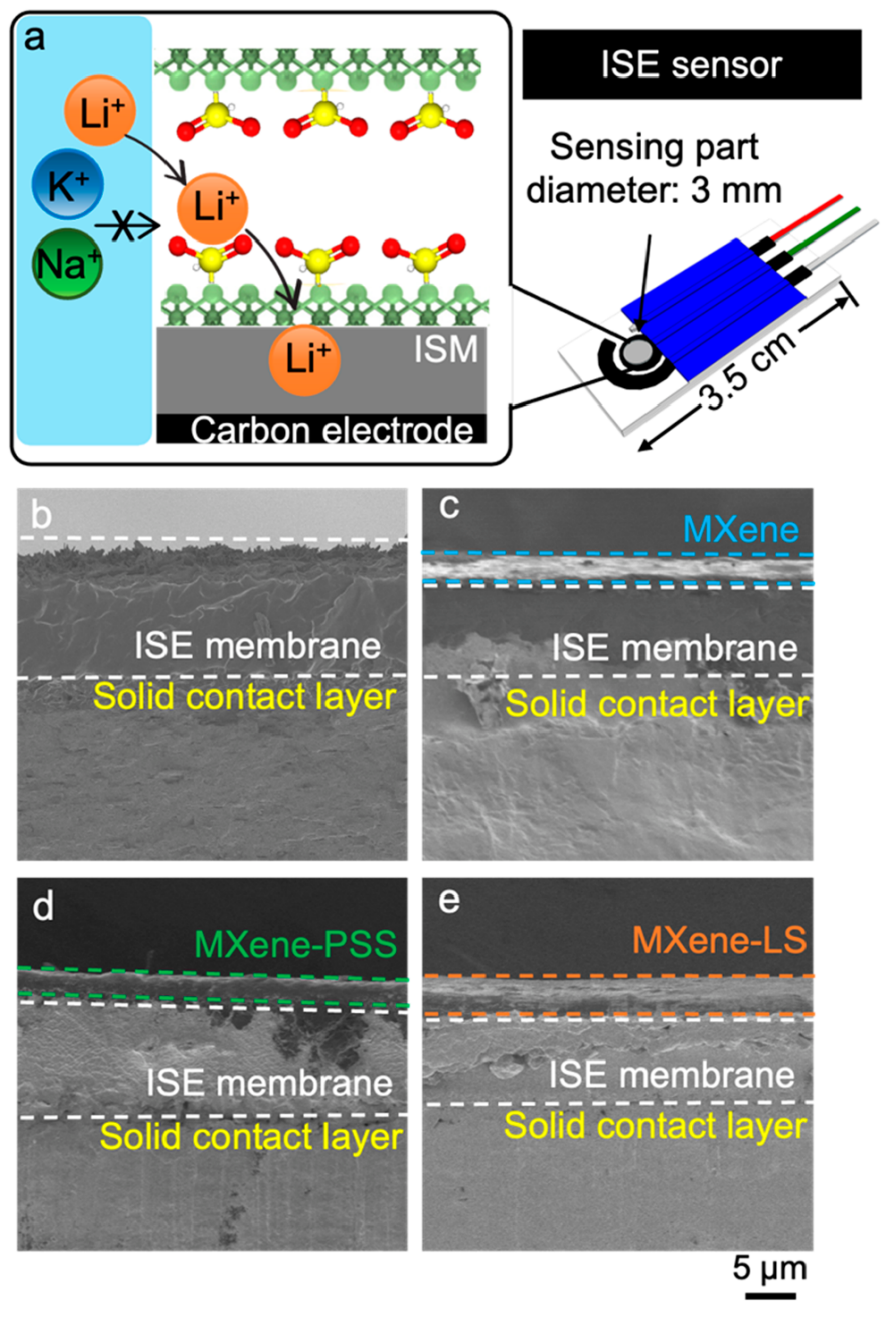
(a) Diagram of the MXene-SO_3_H coated Li^+^ ISE sensor. (b–e) Cross-sectional SEM of no coating, MXene coating, and MXene-SO_3_H coating: (b) conventional Li^+^ ISE sensor; (c) MXene-coated Li^+^ ISE sensor; (d) MXene-PSS coated Li^+^ ISE sensor; (e) MXene-LS coated Li^+^ ISE sensor.

In this study, we present a systematic approach to design an MXene-SO_3_H-coated ISE sensor with an approximate coating thickness of 5 μm, with the aim of achieving superior Li^+^ ion permeability, selectivity, and antifouling properties. To evaluate the sensing performance of the MXene-SO_3_H coating layer, two distinct spacing agents, poly(sodium 4-styrenesulfonate) (PSS) and lignosulfonic acid sodium salt (LS), were integrated into the MXene framework, resulting in the MXene-PSS-coated Li^+^ ISE sensor and the MXene-LS-coated Li^+^ ISE sensor (Figure 1a, Figure S1). Key performance metrics, such as sensor sensitivity (Nernst slope), response time, selectivity, and long-term stability, were evaluated in wastewater conditions. We characterized the major features of the antifouling property of the MXene-SO_3_H layer, including surface hydrophilicity, zeta potential, and surface roughness. Additionally, the sensor performances were evaluated in the simulated lithium recovery process to provide a more comprehensive view of the sensor’s applicability and performance in real-world scenarios.

## Materials and Methods

### Fabrication of MXene/MXene-SO_3_H Nanosheets

MXene (Ti_3_C_2_T_x_) nanosheets were synthesized through selective etching of the aluminum layer from Ti_3_AlC_2_ (MAX phase) precursors. To accomplish this, an in situ hydrofluoric (HF) forming etchant, consisting of lithium fluoride and hydrochloric acid (HCl), was employed, following a slightly modified version of previously established methods.^25^ A comprehensive description of the MXene fabrication process can be found in earlier reports^26^ and Text S1.

Upon obtaining a stable MXene colloidal solution exhibiting a dark green color (Figure S2), it was diluted to a concentration of 1 mg/mL. Poly(sodium 4-styrenesulfonate) (PSS) with an average molecular weight of 70,000 and lignosulfonic acid sodium salt (LS) with an average molecular weight of 52,000 were separately dissolved in deionized (DI) water to create 1 mg/mL solutions. Subsequently, these PSS and LS solutions were mixed with the MXene suspension (Figure S2). The resulting combination was then allowed to equilibrate at room temperature for 24 h to facilitate surface coating formation.

### Fabrication of ISE Sensors

The ISE sensor was fabricated using screen-printing technology (eDAQ, ET083) to create a working electrode (length: 3.5 cm; width: 1.5 cm; thickness: 0.1 cm). To make the sensing membrane, 10 μL of the Li^+^ ionophore polymer mixture was drop-casted onto the working electrode’s surface (diameter: 3 mm). The solid contact layer solution (10 μL) between the membrane and the working electrode surface was made of single-walled carbon nanotubes to enhance the sensor signal stability and reduce the reading drift.^27^ More information on the solid contact and Li^+^ ionophore polymer mixtures cocktail production can be found in Text S2. This conventional Li^+^ ISE sensor was employed as the control sample. To prepare the MXene/MXene-SO_3_H-coated Li^+^ ISE sensors, 20 μL of pristine MXene, MXene-PSS, and MXene-LS solutions were drop-casted onto the surface of the conventional Li^+^ ISE sensor with 8 μL of Li^+^ ionophore polymer mixture, respectively. After being fully dried under vacuum conditions, all of the sensors were stored at 4 °C overnight for further characterization (Figure S3).

### Characterization of MXene/MXene-SO_3_H Membrane Coating

The synthesized conventional Li^+^ ISE sensor and MXene/MXene-SO_3_H-coated Li^+^ ISE sensors were comprehensively characterized using different techniques. The surface and cross-sectional morphology of different membranes were observed by a field-emission scanning electron microscopy (FE-SEM) system (SU8100, Hitachi, Japan). The energy-dispersive X-ray (EDX) test was conducted with the LEO 1530 scanning electron microscope (SEM). The cross-sectional samples were obtained by free-breaking the working electrodes of these ISE sensors after immersing them into liquid nitrogen for around 10 min. The details of the sample preparation and morphology analysis are described in Text S3. The surface elemental analysis was performed by X-ray photoelectron spectroscopy (XPS) measurements using a Thermo ScientificK-α XPS spectrometer (Thermo Fisher Scientific, Waltham, MA). The surface charge of the ISE sensors was determined by ζ-potentials characterized by a ζ-potential analyzer (Zetasizer ZEN 3600 Nano-ZS, Malvern Instruments, U.K.). The contact angle (hydrophilicity) of the sensor surface was examined by measuring the contact angle using a Contact Angle Measurement System (Model 250; Ramé-Hart Instrument Co., Succasunna, NJ, USA). The topography images of the relative surface roughness of the ISE sensors were measured with a Dimension Icon atomic force microscope (AFM, Bruker). The scanning of each sample was carried out over dimensions of 5 μm.

### Characterization of the ISE Sensor Performance

Nernst slope for calibration (mV/dec), response time (s), sensitivity for detection limit (M), and selectivity over Na^+^ and K^+^ were performed to examine the Li^+^ ISE sensor performance with and without the MXene/MXene-SO_3_H-coating. The details of the characterization tests are described in Text S4. The selectivity was quantified by the separate solution method (SSM),^28^ and the experimental details are described in Text S5. The ISE sensors were immersed in 1 ppm LiCl solutions for 48 h prior to each characterization test. This conditioning procedure was demonstrated to be essential for minimizing data drift.^29^ All of the tests were examined in triplicate.

### Long-Term Stability and Antifouling

The long-term accuracy and durability of the MXene-SO_3_H-coated Li^+^ ISE sensors were evaluated and contrasted with those of conventional Li^+^ ISE sensors. The test was conducted in the wastewater collected from a wastewater treatment plant in Buford, GA (chemical oxygen demand: ∼320 mg/L, NH_4_^+^: ∼35 mg/L, Li^+^: <1 mg/L, bacterial count: 10^6^–10^9^ CFU/mL). Additional LiCl was added to the wastewater to increase the Li^+^ concentration to 10 mg/L for better observation. Details of the long-term accuracy tests are described in Text S6. After the long-term test, the bacterial counts on the sensor surface were observed by a fluorescence microscope (Zeiss Axio Observer 7). Details of the fluorescence microscope test are described in Text S7. The electrochemical measurements of the MXene/MXene-SO_3_H-coated Li^+^ ISE sensors were performed at room temperature by using a BASi PalmSens4 potentiostat (PalmSens BV, Houten, Utretch, The Netherlands), in which the membrane-coated electrode, Ag/AgCl electrode, and platinum electrode were used as the working electrode, reference electrode, and counter electrode, respectively. The details of the electrochemical analysis are described in Text S8.

### MXene-SO_3_H-Coated Li^+^ ISE Sensor Application for Lithium Recovery

The fabricated MXene-SO_3_H-coated Li^+^ ISE sensors were submerged into a two-chamber system to examine the sensor accuracy during the lithium recovery process under the interference of Ca^2+^ and Mg^2+^ (Figure S4). The volume of each chamber is 30 mL, and a commercialized nanofiltration membrane (FilmTec NF270-4040, USA) was mounted between two chambers to simulate the Li^+^ diffusion during the lithium recovery process. The diffusion experiments were conducted with simulated brine water as the feedwater, which contained 0.1 M Li^+^, and 0.05 M K^+^, Na^+^, Mg^2+^, and Ca^2+^, respectively. The MXene-SO_3_H-coated Li^+^ ISE sensors were implemented into each chamber, and the sensor readings were recorded using a BASi PalmSens4 potentiostat over 12,000 s. The Li^+^ concentrations obtained by each sensor were validated by inductively coupled plasma-optical emission spectrometry (ICP-OES) (Perkin-Elmer Optima 8000).

## Results and Discussion

### Membrane Preparation and Characterization

Synthesized MXene nanosheets, as revealed in our prior study, exhibit lateral dimensions of several hundred nanometers, with a size distribution ranging from 600 to 1500 nm and an average size of 955 nm.^23^ These substantial lateral dimensions and size distribution contribute to an increased specific surface area, providing a greater number of interaction sites between the MXene nanosheet and lithium ions. This potentially augments the lithium-ion transport from the solute to the ISE membrane surface.^30^ Furthermore, the nanometric thickness of MXene nanosheets ensures diminutive diffusion pathways for lithium ions, which can accelerate the lithium-ion diffusion rate and enhance electron transfer capabilities in electrochemical sensor applications.^31^

Upon drop-casting the MXene, MXene-PSS, and MXene-LS nanosheets onto the ISE surface, additional characterizations were executed on the conventional, MXene-coated, MXene-PSS coated, and MXene-LS coated Li^+^ ISE sensors using XPS, SEM and EDX. The XPS analysis for the MXene-coated Li^+^ ISE sensor revealed an F-Ti peak at 684.38 eV in the F1s spectra, a feature absent in the conventional Li^+^ ISE sensor, which signifies successful MXene nanosheet coating onto the ISE membrane surface (Figure S5). The binding energy of F-Ti for the MXene-PSS coated and MXene-LS coated Li^+^ ISE sensors showed a shift from 684.38 to 684.78 eV, indicative of acid–base pair formation (Figure S5). This implies an interaction between these sites with the protons from SO_3_H groups.^32^ Additionally, the S2p spectra of MXene-PSS-coated and MXene-LS-coated Li^+^ ISE sensors exhibited two distinct peaks between 165 and 170 eV corresponding to S2p_1/2_ and S2p_3/2_, which is a clear indication that a substantial quantity of SO_3_H groups was incorporated into the MXene nanosheets. The successful integration of spacing agents within the MXene interlayer was further confirmed through a comparative analysis of the surface elemental composition across various membranes. The XPS results revealed the presence of titanium and fluorine on the surface of the MXene-coated Li^+^ ISE sensor compared to the conventional Li^+^ ISE sensor (which exhibited zero percentages of titanium and fluorine), further affirming the coating of MXene nanosheets on the ISE membrane surface (Table S2). The presence of sulfur was observed on the surface of MXene-PSS-coated and MXene-LS-coated Li^+^ ISE sensors, yet the MXene-coated Li^+^ ISE sensor exhibited a zero percentage of sulfur, further substantiating the incorporation of spacing agents (SO_3_H groups) into the MXene nanosheets (Table S2).

The successful coating of MXene/MXene-SO_3_H nanosheets was further proven by the SEM and EDX analysis. The side view of the SEM images represented ∼5 μm MXene/MXene-SO_3_H nanosheet layers successfully coated onto the ISE membrane surface and exhibited similar membrane thickness and higher smoothness than conventional Li^+^ ISE surface (Figure 1b–e). The EDX image results of each sensor also showed agreement with the XPS findings. For the MXene-coated Li^+^ ISE sensor, the presence of titanium and fluorine in the EDX images confirmed the successful application of the MXene nanosheet on the sensor surface. Conversely, the sulfur signatures in the EDX images of both the MXene-PSS-coated and MXene-LS-coated Li^+^ ISE sensors indicated the successful integration of spacing agents (SO_3_H groups) within the MXene nanosheets (Figure S6).

### Characterization of the ISE Sensor Performance

**Figure 2:**
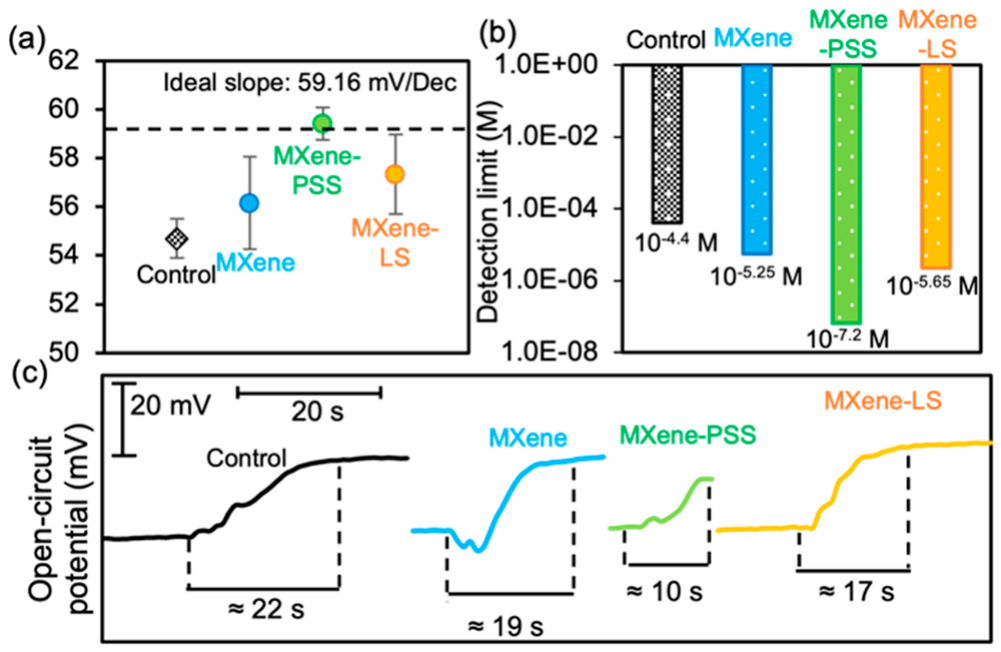
Characterization tests of different Li^+^ ISE sensors: (a) calibration (Nernst slope); (b) sensitivity (detection limit); (c) response time.

The electrochemical properties of the conventional, MXene-coated, and MXene-SO_3_H-coated Li^+^ ISE sensors were comparatively studied at room temperature. According to the Nernst equation, , the conventional Li^+^ ISE sensor displayed a slope of 54.70 mV/dec, which aligns with previous reports.^33,34^ The non-Nernstian behavior of the conventional Li^+^ ISE sensor can be attributed to the nonideality of the membrane’s ion exchange sites inside the PVC-based membrane.^35,36^ Upon coating MXene on the conventional Li^+^ ISE sensor, the slope saw a slight rise to 56.15 mV/dec. Additionally, when the SO_3_H groups were incorporated into the MXene nanosheets, the MXene-SO_3_H-coated Li^+^ ISE sensors showed ideal-Nernst responses, with 59.42 mV/dec for the MXene-PSS-coated Li^+^ ISE sensor and 57.34 mV/dec for the MXene-LS-coated Li^+^ ISE sensor (Figure 2a, Figure S7). In regard to the detection limit (sensitivity), the conventional Li^+^ ISE sensor had a detection limit of 10^–4.4^ M (276.29 μg/L), which decreased to 10^–5.25^ M (39.03 μg/L) with the MXene-coating. This downward trend in detection limits was even more noticeable among the MXene-SO_3_H-coated Li^+^ ISE sensors, with the MXene-PSS-coated Li^+^ ISE sensor showcasing the lowest limit of 10^–7.2^ M (0.44 μg/L) and the MXene-LS-coated Li^+^ ISE sensor displaying a limit of 10^–5.65^ M (15.54 μg/L) (Figure 2b, Figure S8). The response time of the conventional Li^+^ ISE sensor was 22 s, slightly longer than the MXene-coated Li^+^ ISE sensor’s 19 s. The MXene-SO_3_H-coated Li^+^ ISE sensors showed faster response times, 10 s for MXene-PSS-coated and 17 s for MXene-LS-coated Li^+^ ISE sensors (Figure 2c). Furthermore, we determine the selectivity coefficients (*K*_Li^+^,Na^+^_^pot^ and *K*_Li^+^,K^+^_^pot^) of the Li^+^ ISE sensors using the separate solution method (SSM) method (Text S5). When compared to the conventional Li^+^ ISE sensor, the MXene-coated Li^+^ ISE sensor showed higher log*K*_Li^+^,Na^+^_^pot^ and log*K*_Li^+^,K^+^_^pot^ values across all concentration ranges from 3.6 × 10^–5^ to 1.8 × 10^–2^ M (e.g., at 1.8 × 10^–2^ M, the selectivity coefficients of the MXene-coated Li^+^ ISE sensor were log*K*_Li^+^,Na^+^_^pot^: −1.43, log*K*_Li^+^,K^+^_^pot^: −1.90, while the selectivity coefficients of the conventional Li^+^ ISE sensor were log*K*_Li^+^,Na^+^_^pot^: −2.26, log*K*_Li^+^,K^+^_^pot^: −2.47) (Tables S3, S4), suggesting a compromise in the Li^+^ selectivity for MXene-coated Li^+^ ISE sensor. Interestingly, the log*K*_Li^+^,Na^+^_^pot^ and log*K*_Li^+^,K^+^_^pot^ values of the MXene-SO_3_H-coated Li^+^ ISE sensors were even lower than the conventional Li^+^ ISE sensor (e.g., at 1.8 × 10^–2^ M, the selectivity coefficients of MXene-PSS Li^+^ ISE sensor were log*K*_Li^+^,Na^+^_^pot^: −2.37, log*K*_Li^+^,K^+^_^pot^: −2.54, and the selectivity coefficients of MXene-LS Li^+^ ISE sensor were log*K*_Li^+^,Na^+^_^pot^: −2.51, log*K*_Li^+^,K^+^_^pot^: −2.51) (Tables S3, S4), indicating enhanced selectivity with this modification.

The results of our sensor characterizations indicated that MXene nanosheets significantly enhance the performance of Li^+^ ISE sensors across multiple parameters, including the Nernst slope, detection limit (sensitivity), and response time. This improvement can be attributed to the distinctive properties of MXene, namely high conductivity and rapid ion transport capabilities.^37^ The exceptional electronic conductivity and layered structure of MXene nanosheets facilitate rapid ion transport and efficient charge transfer,^38^ which is crucial for minimizing response time and amplifying detection limits in ISE sensors. Moreover, the flexible interlayer spacing of MXene, which lends itself to tunability, permits the intercalation of various organic and inorganic ions, thereby increasing its affinity for specific ions, such as Li^+^.^39^ The decreased selectivity of Li^+^ over Na^+^ and K^+^ (i.e., higher logK_Li^+^,Na^+^_^pot^ and logK_Li^+^,K^+^_^pot^ values) in the MXene-coated Li^+^ ISE sensor could be defined by the Eisenman Sequence I, which denotes the alkali metal ion selectivity sequence of K^+^ > Na^+^ > Li^+^ among the MXene nanosheets.^40^ Interestingly, the MXene-SO_3_H membrane exhibited Eisenman Sequence XI, denoting a selectivity sequence of Li^+^ > Na^+^ > K^+^. The different behavior of the MXene-SO_3_H coated Li^+^ ISE sensors is largely attributed to the introduction of SO_3_H groups, since the lower binding energies between SO_3_H and Li^+^ indicated a lower energy barrier that the ion needs to overcome during transport. The integration of SO_3_H groups plays a pivotal role in this increased selectivity and provides valuable insights for improving the design and performance of Li^+^ selective electrode sensors.

### Electrochemical Analysis of the ISE Sensors

**Figure 3:**
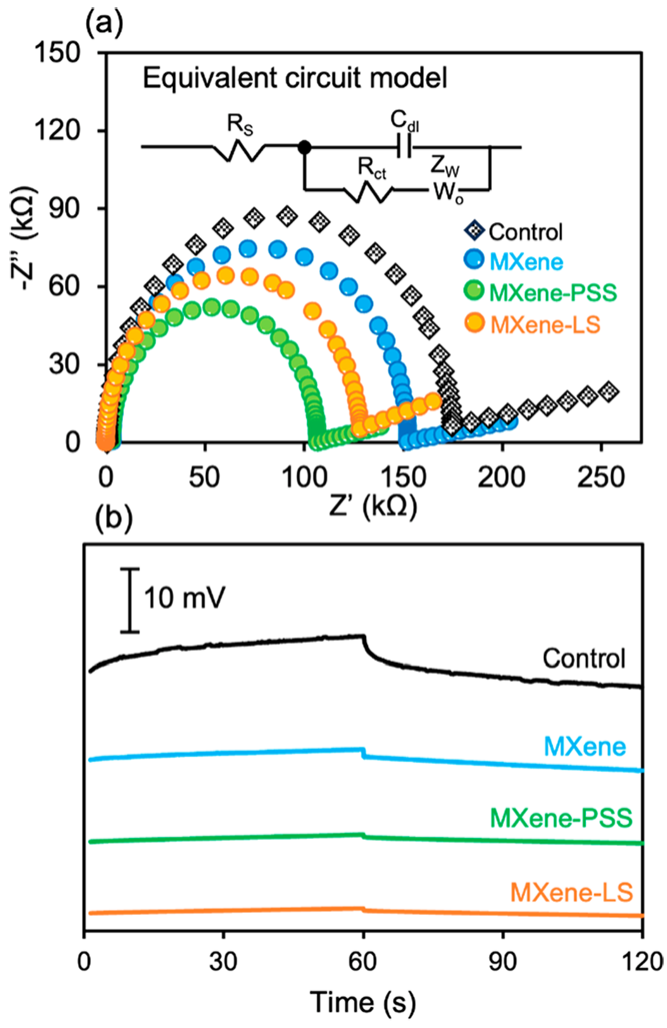
Electrochemical evaluation of different Li^+^ ISE sensors. (a) Impedance analysis with corresponding equivalent circuit diagram models. (b) Chronopotentiometry test.

The electrochemical impedance spectroscopy (EIS) was used to investigate the ion-to-electron transduction of the Li^+^ ISE sensor. As shown in Figure 3a, the impedance plots covered a range from high to low frequencies and a corresponding equivalent circuit was provided for all Li^+^ ISE sensors. Consistent with previous reports, the dominant feature across all Li^+^ ISE sensors was a single semicircle, representing the bulk resistance of the ISE membrane (noted as *R*_s_ in the equivalent circuit model, Figure 3a).^41^ Of note, with the similar membrane thickness (Figure 1b,c), the MXene-coated Li^+^ ISE sensor displayed a lower charge-transfer resistance (*R*_ct_, represented in the equivalent circuit model, Figure 3a) of 152.5 kΩ, compared to the conventional Li^+^ ISE sensor, which displayed a resistance of 169.9 kΩ, suggesting that the MXene coating establishes conductive pathways to enhance the electron transfer process and facilitate ion-to-electron conversion within the membrane.^42^ Interestingly, the MXene-SO_3_H coated Li^+^ ISE sensors exhibited the lowest *R*_ct_ values (106.9 kΩ for the MXene-PSS-coated Li^+^ ISE sensor and 128.4 kΩ for the MXene-LS-coated Li^+^ ISE sensor, Figure 3a). The enhanced performance of these sensors can be attributed to the introduction of sulfonic acid (SO_3_H) groups. These groups not only offer additional ion-exchange sites to promote ion-to-electron conversion but also enhance interactions with Li^+^ ions due to their pronounced polar properties, thus facilitating Li^+^ transport across the interface.^43^

Further, the potential stability of the Li^+^ ISE sensors was studied by a constant current chronopotentiometry method, in which a current of ±1 nA was applied and electrode polarization would cause potential decaying. The rationale behind this experiment is to challenge the ISE sensors with the external current of alternating ±1 nA and to assess the reading drift over time () of the sensor.^44^ As shown in Figure 3b, a significant potential decaying of 129.5 μV/s was observed in the conventional Li^+^ ISE sensor, showing a similar result as the previous report.^45^ In contrast, the potential decaying exponents for MXene-coated and MXene-SO_3_H-coated Li^+^ ISE sensors were reduced to 53.9 μV/s (MXene), 22.7 μV/s (MXene-PSS), and 19.6 μV/s (MXene-LS), confirming the superior properties of the MXene-coated and MXene-SO_3_H-coated layer.

It should be noted that compared to the MXene-LS-coated Li^+^ ISE sensor, the MXene-PSS-coated Li^+^ ISE sensor exhibited better sensor characterization and electrochemical performance (higher slope, lower detection limit, faster response time, lower impedance, and similar potential drift). The reason for the superior performance of the MXene-PSS-coated Li^+^ ISE sensor can be attributed to the higher concentration of SO_3_H groups on the MXene-PSS nanosheets (1.38% sulfur) compared to the MXene-LS nanosheets (0.86% sulfur) as demonstrated by the XPS results (Table S2). PSS is a fully synthetic polymer that has a regular structure and high density of SO_3_H groups, which facilitates the addition of these groups onto the surface of MXene nanosheets during the fabrication process.^46^ The higher density of these groups on MXene-PSS could lead to more efficient ion transport and better overall performance than MXene-LS. Therefore, the MXene-PSS coated Li^+^ ISE sensor was selected for further long-term test and lithium recovery test in this study.

### Long-Term Continuous Monitoring of Li^+^ in the Wastewater

**Figure 4:**
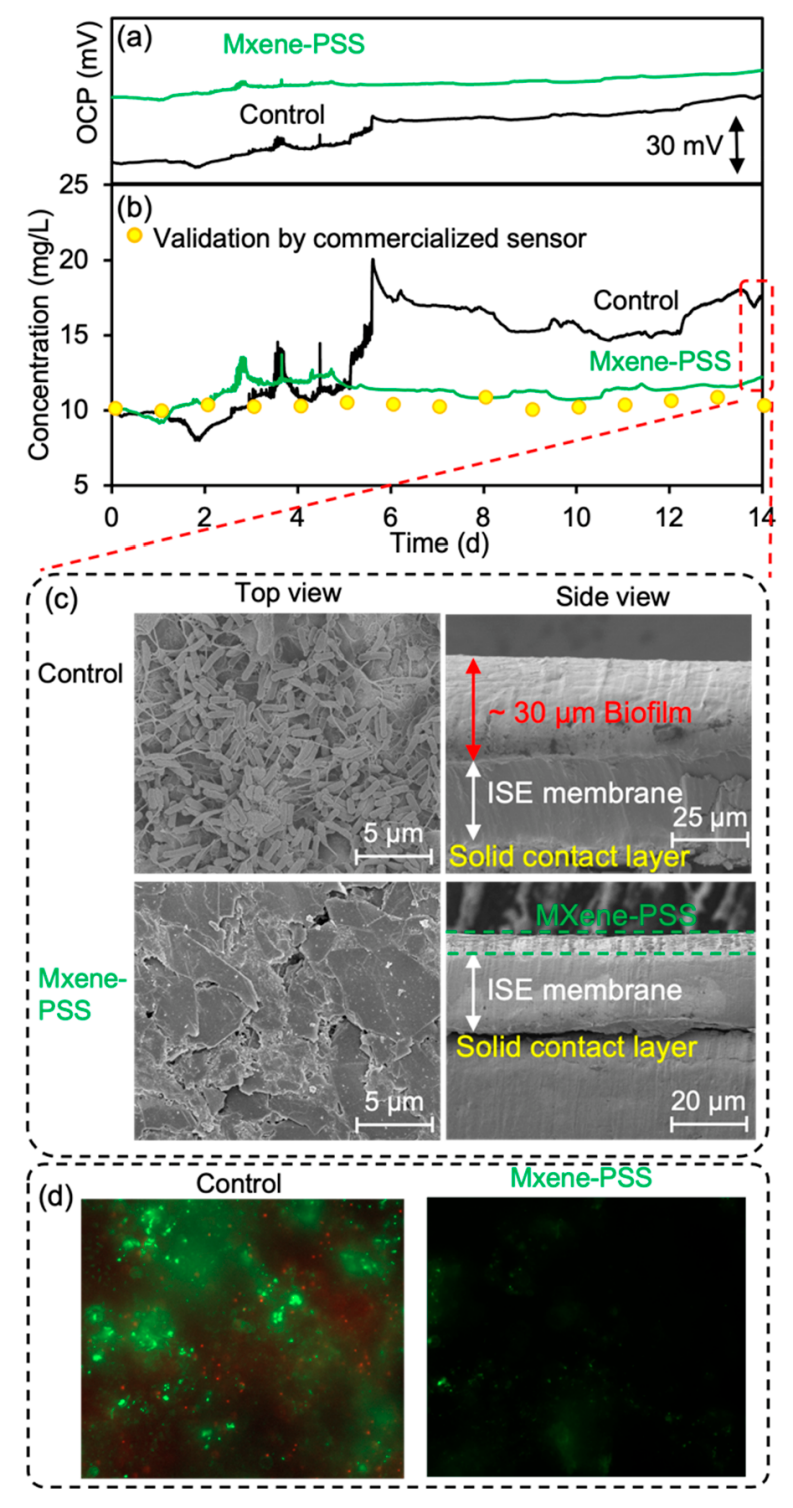
Long-term continuous tests of the conventional Li^+^ ISE sensor (black line) and the MXene-PSS-coated Li^+^ ISE sensor (green line). (a) Potential readings. (b) Concentration readings based on updated daily calibration curves. The yellow circle represents the real concentration obtained by a commercialized Li^+^ ISE sensor. (c) The top view and the cross-sectional view of the SEN images of the conventional Li^+^ ISE sensor and the MXene-PSS coated Li^+^ ISE sensor 14 days in wastewater. (d) Fluorescence microscope images of PI and SYTO 9-incubated bacteria on the conventional Li^+^ ISE sensor (left) and the MXene-PSS coated Li^+^ ISE sensor (right) surface after 14 days in wastewater.

A direct comparison was undertaken to evaluate the long-term accuracy and durability of conventional Li^+^ ISE sensors and MXene-PSS-coated Li^+^ ISE sensors in the wastewater collected from a treatment plant in Buford, GA, which exhibited a chemical oxygen demand of approximately 320 mg/L. To facilitate clearer observations, the Li^+^ concentration within this wastewater was adjusted to around 10 mg/L. When assessing open circuit potential (OCP) readings, the MXene-PSS-coated Li^+^ ISE sensor demonstrated reduced reading drift (1.05 mV/day) in comparison to its conventional counterpart (2.58 mV/day). Additionally, the conventional sensor exhibited a substantial deviation in OCP readings (around 16 mV/h) after six days within the wastewater environment (Figure 4a).

In order to address the sensor reading drift, the OCP readings gathered from the Li^+^ ISE sensors were converted to Li^+^ concentration (mg/L) through the utilization of daily updated calibration curves based on the Nernst equation over the course of 14 days. Verifications of the Li^+^ concentrations in the wastewater were conducted daily using a commercially available Li^+^ ISE sensor. Despite these recalibration efforts, a sizable discrepancy (average error of 37.49%) was observed between the readings of the conventional Li^+^ ISE sensor and the concentration value verified by the commercial sensor (Table S5, Figure 4b), suggesting that the sensor lifespan had been compromised due to biofouling. Conversely, concentration readings from the MXene-PSS coated Li^+^ ISE sensor remained stable with an average error below 10% (Table S5, Figure 4b).

Historically, studies have utilized ideal conditions, such as a 0.1 M LiCl solution, to evaluate reading drift in long-term applications of ISE sensors.^29,47^ Yet, when monitoring wastewater, these sensors are subjected to an array of suspended particles and bacteria, leading to biofouling and subsequently impairing sensor accuracy. These findings were substantiated through SEM imaging. After 14 days of immersion in the wastewater, a significant amount of bacterial cells adhered to the surface of the conventional Li^+^ ISE sensor, forming a biofilm approximately 30 μm thick (Figure 4c). This undoubtedly disrupted the permeation of Li^+^ ions from the bulk wastewater to the sensor surface, impairing accuracy and confirming the discrepancy in concentration readings from the conventional sensor. On the other hand, no biofilms were observed on the surface of the MXene-PSS coated Li^+^ ISE sensor following 14 days of immersion in wastewater, with the MXene-PSS coating remaining predominant on the sensor surface (Figure 4c). This highlights the superior antifouling characteristics of the MXene-PSS coating.

To further investigate the antifouling property of the MXene-PSS coating, SYTO 9 and propidium iodide (PI) colored bacteria on the sensor surface after 14 days in wastewater were observed under a fluorescence microscope. PI can only permeate cells that have lost membrane integrity and develop a red color fluorescence,^48^ while SYTO 9 is a green-fluorescent stain that penetrates both viable and nonviable cells, thus binding to the nucleic acids inside and making them fluoresce green under appropriate illumination.^49^ For the conventional Li^+^ ISE sensor surface, the pronounced presence of green fluorescent cells showed an active microbial population (Figure 4d). In tandem with this, the scattered red fluorescent cells provided evidence of compromised or dead cells within the microbial community. This coexistence of live and dead cells is a hallmark of biofilm maturation, indicative of a biofilm that has been evolving over an extended period on the surface of the conventional Li^+^ ISE sensor. By contrast, the surface of the MXene-PSS coated Li^+^ ISE sensor revealed minimal green fluorescent cells (Figure 4d). The near absence of these green cells, combined with the complete lack of red fluorescent cells, strongly suggested no active microbial colonization or resultant biofilm formation. Such findings underscore the enhanced anti-biofouling capabilities of the MXene-PSS-coated Li^+^ anti-biofouling ISE sensor.

### Evaluation of the Antibiofouling Property of the MXene-SO_3_H Membrane Coating

**Figure 5:**
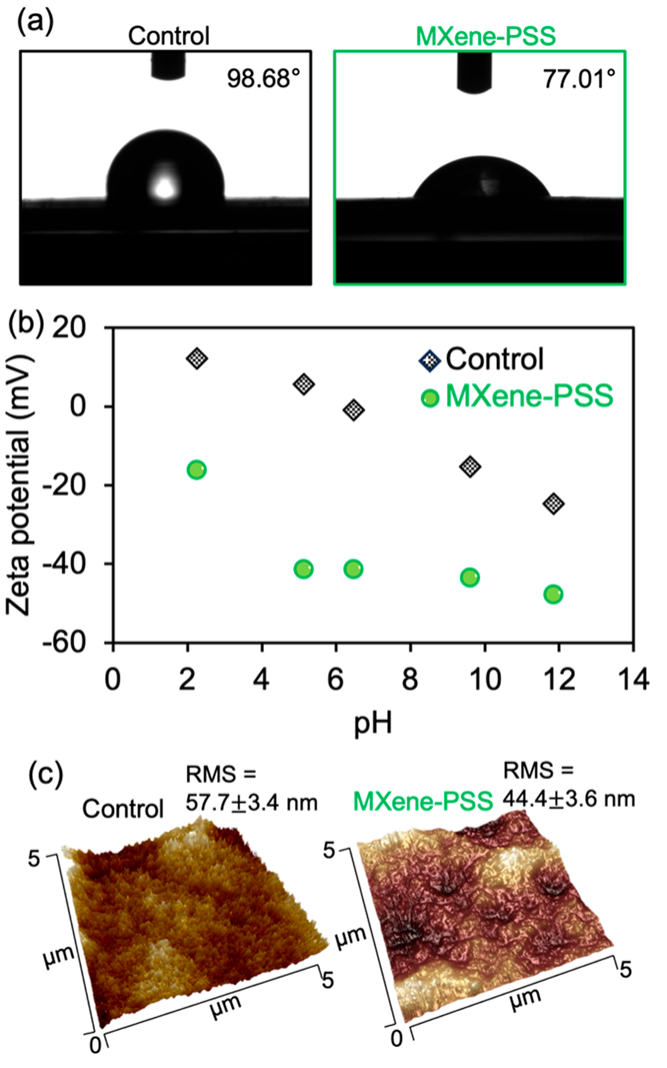
Characterization of the MXene-PSS coating surface. (a) The contact angle test for the conventional Li^+^ ISE sensor (left) and the MXene-PSS coated Li^+^ ISE sensor (right). (b) ζ potential of the conventional Li^+^ ISE sensor (black dots) and the MXene-PSS-coated Li^+^ ISE sensor (green dots) as a function of pH. (c) The corresponding AFM root-mean-square (RMS) roughness of the conventional Li^+^ ISE sensor (left) and the MXene-PSS coated Li^+^ ISE sensor (right).

We propose that the outstanding antifouling capabilities of the MXene-PSS coating can be attributed to three primary factors. First, the hydrophilic nature of the MXene-PSS surface deters adhesion by hydrophobic foulants, including organic compounds and bacteria.^50^ To validate this hypothesis, we observed the contact angle to characterize the hydrophilic surface rendered by the MXene-PSS coating. The contact angle of the MXene-PSS coating layer was 77.01°, which was more hydrophilic than the conventional Li^+^ ISE sensor (98.68°, Figure 5a). The highly hydrophilic surface of the MXene-PSS helps create a hydration layer over the surface, acting as a physical and energetic barrier against the adhesion of foulants.^51^

Second, the electrostatic interactions between the ISE sensor surface and the constituents of wastewater are indeed a crucial determinant of the sensor’s long-term durability. This is predominantly due to the electrostatic repulsion that occurs between the negatively charged surface of the MXene-PSS-coated ISE sensor and similarly charged entities in wastewater such as microbial cells. The negative surface charge on the MXene-PSS coating layer arises from the incorporation of SO_3_H groups from the PSS polymer.^52^ It is essential to recognize that, despite inherent variations in the physical and chemical structure of bacterial surfaces, most bacteria—and indeed, many natural surfaces—are predominantly negatively charged.^53^ This makes the negatively charged MXene-PSS coating particularly effective in repelling the common microbial contaminants found in wastewater. The zeta potential (ζ-potential) of the MXene-PSS coated Li^+^ ISE sensor demonstrated a more pronounced negative surface charge (−16.1 to −47.8 mV) compared to the conventional Li^+^ ISE sensor (12.2 to −24.6 mV), spanning pH values of 2.3 to 11.9 (Figure 5b). At pH 6.48, the ζ-potential of the MXene-PSS-coated Li^+^ ISE sensor was recorded at −41.3 mV, echoing the findings from our prior study where we utilized polytetrafluoroethylene (PTFE) as an antifouling material in ISE sensors, which yielded a ζ-potential of −43.7 mV at pH 7.^54^ This heightened electrokinetic repulsion of negatively charged microbial cells in wastewater, demonstrated by the MXene-PSS coated Li^+^ ISE sensor, provides a compelling explanation for its superior antifouling performance.

Lastly, the MXene-PSS coating creates a smooth, uniform surface layer, considerably reducing surface irregularities and roughness. Previous studies have shown that bacteria are more likely to attach to rough surfaces compared to smoother ones.^55^ As such, the smooth surface delivered by the MXene-PSS coating offers fewer attachment points for foulants, thereby bolstering the fouling resistance. For conventional ISE sensors, the membrane surface can contain a variety of impurities depending on its synthesis and purification processes. These impurities can include residual monomers, additives, or plasticizers, which are often used to enhance certain properties of the PVC, such as flexibility.^56^ When PVC is used to form a membrane for an ISE sensor, these impurities can result in an uneven distribution of material on the surface, leading to a less smooth membrane surface (Figure 1b). Furthermore, the process of preparing the PVC membrane, often involving a phase inversion process with PVC dissolution in a solvent (e.g., tetrahydrofuran) and subsequent precipitation, can also contribute to surface roughness and irregularities.^57^ The corresponding AFM image showed a root-mean-square (RMS) roughness of 57.7 ± 3.4 nm for the conventional Li^+^ ISE sensor surface (Figure 5c). Conversely, the MXene-PSS coating, composed of 2D nanosheets, can form a more uniform and smoother layer on the ISE sensor surface (Figure 1e). The corresponding AFM image depicted a smoother surface with an RMS roughness of 44.4 ± 3.6 nm for the MXene-PSS-coated Li^+^ ISE sensor surface (Figure 5c), comparable to previous antifouling coatings such as poly(vinylidene fluoride) (PVDF)/graphene oxide (GO) ultrafiltration membranes (52 nm^58^) and GO-TiO2/PVC matrix (39.4 nm^59^). This impressive resistance to biofouling results from the combined influence of a hydrophilic surface, a strong negative surface charge over the pH range typical in municipal wastewater (pH: 6–9), and the fine nanoscale surface roughness offered by the MXene-PSS coating.

## Significance and Future Perspective

In this study, we designed a MXene-SO_3_H-coated Li^+^ ISE sensor that exhibited superior Li^+^ ion permeability and selectivity. Our comparative analyses demonstrated the distinct advantages of the MXene-PSS-coated Li^+^ ISE sensor over its conventional counterparts. The MXene-PSS coating led to a higher slope, lower detection limit, faster response time, enhanced Li^+^ selectivity, and reduced impedance. This heightened performance can largely be attributed to the abundant SO_3_H groups on the MXene-PSS nanosheets, which facilitated efficient ion transport and improved overall sensor functionality. Notably, the MXene-PSS-coated Li^+^ ISE sensor also showed remarkable durability and antifouling properties when tested in a real-world wastewater environment, with an average Li^+^ concentration reading error of less than 10% compared to the commercialized sensors, outperforming the conventional Li^+^ ISE sensor. The key factors contributing to the antifouling properties were attributed to the hydrophilic nature of the MXene-PSS surface, the negative surface charge imparted by the presence of SO_3_H groups, and the smooth and continuous surface layer that minimized surface irregularities.

The fabricated MXene-PSS Li^+^ ISE sensors were submerged in the two-chamber system to test the sensor performance during the lithium recovery process. Both sensors on the feedwater side and the permeate water side displayed exemplary performance, accurately capturing the lithium concentration changes under the interference of other ions (Figure S9). The promising results showcase the potential of our developed MXene-PSS Li^+^ ISE sensor as a crucial tool for lithium recovery. Real-time and accurate monitoring of lithium concentration gradients is essential during the recovery process. By ensuring that lithium recovery operations are conducted under optimal conditions, we can reduce energy consumption and achieve cost savings. This could lead to a decreased cost per unit of lithium recovered, ultimately making the lithium recovery process more competitive and sustainable in the future.

However, the traditional drop-casting method used for coating MXene and MXene-SO_3_H nanosheets onto the Li^+^ ISE sensor surface might introduce variability in the coating thickness and uniformity. Employing more uniform coating strategies, such as electrospray, could potentially enhance sensor performance by ensuring a consistent interaction between the Li^+^ ions and the MXene-PSS coating. Therefore, future studies should investigate refining the coating process with controlled deposition parameters or exploring self-assembly techniques.

## Supporting Information Available

Synthesis of MXene nanosheet; Li^+^ ionophore cocktail and solid contact fabrication; morphology analysis; characterization test; selectivity test; long-term stability test; fluorescence microscope test; electrochemical analysis; chemical structure and 3D molecular structures PSS and LS; photograph of the MXene, MXene-PSS, and MXene-LS suspensions; photograph of the Li^+^ ISE sensors; schematic illustration of the experimental setup for lithium recovery; XPS spectra; EDX images of the sensor surface; calibration curves; detection limit (sensitivity) test; MXene-PSS Li^+^ ISE sensor readings under the simulated brine water recovery process; concentration of each ion in the typical water/wastewater streams for lithium recovery; surface chemical compositions (data from XPS); selectivity coefficients; Li^+^ concentrations in wastewater (PDF)
