# Supplementary material for: MXene-Coated Ion-Selective Electrode Sensors for Highly Stable and Selective Lithium Dynamics Monitoring
Source: Environ Sci Technol. 2023 Dec 11;58(2):1359–68. doi: 10.1021/acs.est.3c06235 (PMC10795166; doi:10.1021/acs.est.3c06235)
Supplement: Supplementary file 1 — es3c06235_si_001.pdf [file es3c06235_si_001.pdf]

Supporting Information for

MXene-Coated Ion-Selective Electrode Sensors for Highly Stable and Selective Lithium Dynamics Monitoring

Yuankai Huang[a†], Moyosore A. Afolabi[a†], Lan Gan[a], Su, Liu[a], Yongsheng Chen[a,*]

[a] School of Civil and Environmental Engineering, Georgia Institute of Technology, Atlanta, Georgia 30332, United States

(*corresponding author: Yongsheng Chen, Email: yongsheng.chen@ce.gatech.edu, Phone: 4048943089)

[†]These authors contribute equally to this paper.

Number of pages: 25

Number of texts: 8

Number of figures: 9

Number of tables: 5

Text S1 The Synthesis of MXene Nanosheet

A solution of 9 M hydrochloric acid (HCl) was prepared by combining 15 mL of 12 M HCl with 5 mL of 18.2 MΩ-cm deionized water, yielding a total volume of 20 mL. This mixture was placed in a high-density polyethylene (HDPE) bottle and stirred at 100 rpm for 5 minutes. Subsequently, 1.6 g of lithium fluoride was dissolved in the acidic solution and stirred for 30 minutes. Commercial Ti_3AlC_2 (MAX) powder (1 g) was gradually added to the solution over a period of 5 minutes. The HDPE bottle was then positioned and secured in a water bath maintained at 35°C and subjected to magnetic stirring at 300 rpm for 24 hours. The mixture underwent multiple centrifugation cycles (3,500 rpm x 10 min) with 18.2 MΩ-cm deionized water to wash it until the supernatant's pH reached approximately 6. The multilayer MXene suspension was sonicated under argon for 30 minutes, after which unreacted MAX sediment was removed through centrifugation at 3,500 rpm for 1 hour. The resulting MXene suspensions were stored at 4°C for subsequent processing.

Text S2 Li^+ Ionophore Cocktail and Solid Contact Fabrication

Li^+ Ionophore Cocktail. This cocktail consisted of known Lithium ionophore VII (2-(6-dodecyl-1,4,8,11-tetraoxacyclotetradec-6-yl)ethyl diethyl phosphate, 7% weight by weight, w/w Sigma-Aldrich), polyvinyl chloride (PVC, 30% w/w, Sigma-Aldrich, to reduce the mobility of the ionophores and additives inside the membrane[1]), plasticizer 2-nitrophenyloctylether (2-NPOE, 60% w/w, Sigma-Aldrich, to maintain the proper physical and mechanical properties of the membrane[2]), and additives potassium tetrakis[3,5-bis(trifluoromethyl)phenyl]borate (3% w/w, to improve the binding ability of the membrane). The Li^+ ionophore cocktail (100 mg) was fully dissolved in 500 μL of tetrahydrofuran (THF, ⩾99.5%, Sigma-Aldrich, served to dissolve the membrane). The dispersion was homogenized by using a sonicator for 15 min. The mixture was stored at 4°C for future use.

Solid Contact. Single-walled carbon nanotubes (SWCNTs, >98%, Sigma-Aldrich) were used as a solid contact layer. The SWCNTs were deposited by drop-casting an aqueous dispersion (0.2% w/w) and sodium dodecyl sulfate (SDS, 1% w/w, Sigma-Aldrich) onto the working electrode surface (10 μL). The dispersion was homogenized by using sonicator for 30 min. The solid contact layer was left overnight at room temperature to dry out.

Text S3 Morphology Analysis

Prior to Scanning Electron Microscopy (SEM) analysis, fixation, and dehydration procedures are necessary for wastewater-treated ISE sensors with a bacterial presence on the surface.

Fixation. The working electrode surfaces were thoroughly rinsed with phosphate-buffered saline (PBS, Sigma-Aldrich) solution three times to remove any loose debris. Fixation was performed by submerging the working electrodes in a 0.1 M phosphate buffer (Sigma-Aldrich), pH 7.4, containing 2.5% glutaraldehyde (Sigma-Aldrich) and allowing them to remain overnight at 4°C. The following day, the samples were removed from the buffer and rinsed three times with phosphate buffer, each time for 20 minutes at room temperature.

Dehydration. Following the fixation process, the working electrodes containing fixed bacteria were rinsed with 18.2 MΩ-cm deionized water three times, each time for 10 minutes. Subsequently, the samples were rinsed in a series of graded ethanol (Sigma-Aldrich) solutions: 30%, 50%, 75%, 95% (once), and 100% (four times), each time for 15 minutes. The samples were then rinsed with a mixture of 50% Hexamethyldisilazane (HMDS, Sigma-Aldrich) and 50% ethanol, followed by a mixture of 75% HMDS and 25% ethanol (once), and finally, 100% HMDS (twice), each time for 30 minutes. The samples were left to dry in Petri dishes with the lids partially closed in a fume hood overnight. All rinsing procedures were conducted in 10 mL glass bottles secured on a New Brunswick™ Innova® 2100 platform shaker, with a rotation rate of 80 rpm at room temperature.

SEM Analysis. Both surface and cross-section morphology of the ISE sensors was characterized using a scanning electron microscopy (FE-SEM) system (SU8100, Hitachi, Japan). The sensor samples were coated with a 15 nm gold‒palladium layer (Cressington 108A carbon coater, Quorum Q-150 T ES) prior to the analysis.

Energy Dispersive X-ray (EDX) Analysis. The EDX test was performed to provide elemental composition information on the sensor surface. The EDX was conducted by the LEO 1530 scanning electron microscope (SEM). The sensor samples were coated with a ~50 nm carbon layer (Cressington 108A carbon coater, Quorum Q-150 T ES) prior to the analysis.

Text S4 Characterization Test of the Li^+ ISE Sensors

The potentiometric measurements were carried out using a BASi PalmSens4 potentiostat (PalmSens BV, Houten, Utretch, The Netherlands) at room temperature with Ag/AgCl (3 M KCl) as the reference electrode. The detection limits (sensitivity) of the sensors were determined by starting with the lithium concentration of 1×10^{-7} M, followed by recording the potential readings in incremental concentrations (10^{-8} to 10^{-2} M lithium as Li^+ solution). The response time was identified by measuring the length of time at which the open circuit potential (OCP) values became equal to their steady-state value within 1 mV or reached 90% of the final value when Li^+ shocks were introduced in the Nernst slope test.

Text S5 Selectivity Test of the Li^+ ISE Sensors

The selectivity coefficient was determined by measuring the OCP values (mV) of the Li^+ ISE sensor and MXene/MXene-SO_3H-coated Li^+ ISE sensors with the presence and absence of nontargeted interfering ions (K^+, Na^+). Specifically, The OCP (mV) readings of each sensor were recorded under a series of primary ion (Li^+) solutions (0.25 - 128 mg/L) and interfering ion (K^+, Na^+) solutions (0.25 - 128 mg/L), respectively. After obtaining the calibration curves of each sensor with the primary ion and interfering ions, the selectivity coefficient is calculated by using separate solution method (SSM)[3]:

$$log\ k_{A,B}^{pot} = \frac{(E_B - E_A)z_A F}{RTln10} + \left(1 - \frac{z_A}{z_B}\right) log a_A$$

Where E_A and E_B are the OCP values of primary and interfering ions, and z_A and z_B are the ion charges of primary and interfering ions. The selectivity coefficients are calculated at different concentrations (0.25 - 128 mg/L).

Text S6 Long-term Stability Test

Long-term accuracy and durability of the MXene/MXene-SO_3H-coating Li^+ ISE sensors were examined and compared with those of the conventional Li^+ ISE sensors by submerging each type of sensor into a parafilm-sealed beaker containing 300 mL of wastewater collected from the F. Wayne Hill Water Resources Center located at Buford, Georgia. The test period was 14 days, during which one Li^+ shock was introduced into the beaker on the 13th day, to examine the accuracy of these sensors under drastic changes of lithium concentrations in waste streams. The open-circuit potential (OCP) values of each sensor were individually recorded every 30 s using a BASi PalmSens4 potentiostat (PalmSens BV, Houten, Utretch, The Netherlands). Subsequently, all 40,320 OCP readings (mV) per sensor were converted to the Li^+ concentration (mg/L) using the updated daily calibration curves throughout 14 days. In addition, the Li^+ concentrations obtained by each type of sensor were validated against a commercialized Li^+ ion selective electrode sensor.

Text S7 Fluorescence Microscope Test

After the long-term test, the bacterial counts on the sensor surface were observed by a fluorescence microscope (Zeiss Axio Observer 7). SYTO™ 9 Green Fluorescent Nucleic Acid Stain and propidium iodide (PI) were used as fluorophores to test alive and dead cells, respectively. The cell images were captured via the Differential Interference Contrast (DIC) channel. PI was excited at 555 nm (for red fluorescence), SYTO™ 9 was excited at 488 nm (for green fluorescence). All emission light was filtered by a 90 HS filter.

Text S8 Electrochemical Analysis

Impedance Analysis: The effect of MXene/MXene-SO_3H addition on the impedance of the ISE sensor membrane was measured by Electrochemical Impedance Spectroscopy (EIS) techniques. For EIS measurement, the working electrodes of each ISE sensor, a platinum counter electrode and a silver/silver chloride double-junction reference electrode were inserted into a conventional three-electrode cell containing 0.1 M LiCl solution. The results were recorded in the frequency range from 100 kHz to 0.1 Hz with an excitation amplitude of 10 mV by using a BASi PalmSens4 potentiostat (PalmSens BV, Houten, Utretch, The Netherlands). Data were fitted based on the equivalent circuit models in the ZView software.

Chronopotentiometry Test: For chronopotentiograms, a positive constant current of 1 nA was applied for 60 s, followed by a negative constant current of 1 nA for 60 s. The water layer test was conducted in 0.1 M LiCl, 0.1 M NaCl, and then 0.1 M LiCl aqueous solutions.

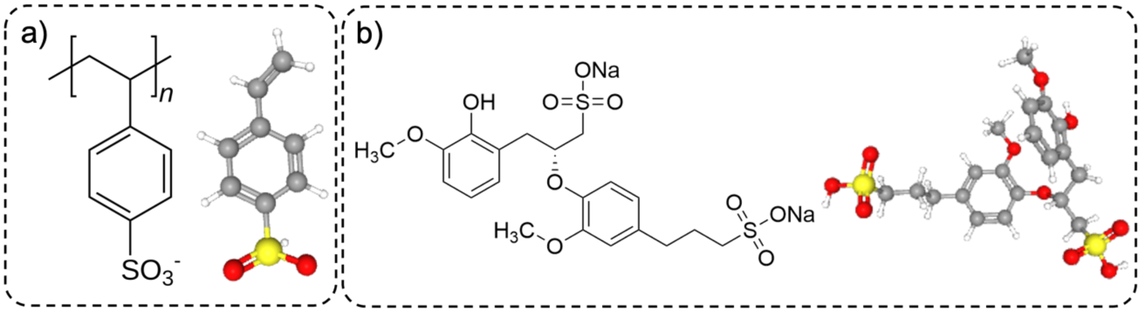

Figure S1. The chemical structure and 3D molecular structures of (a) poly (styrene sulfonic acid sodium salt) (PSS) and (b) lignosulfonic acid sodium salt (LS).

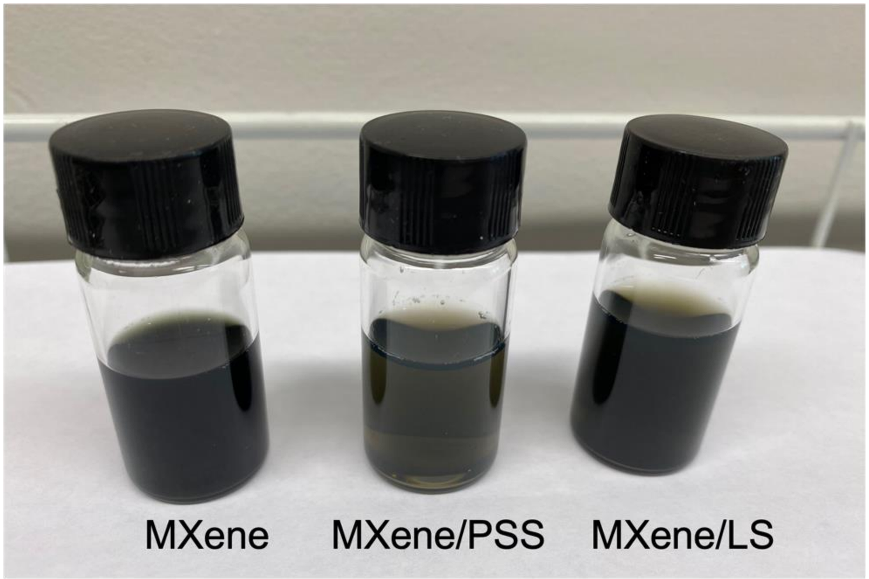

Figure S2. Photograph shows the MXene, MXene-PSS and MXene-LS suspensions. The mass ratio of MXene and the spacing agents is maintained at 1:1.

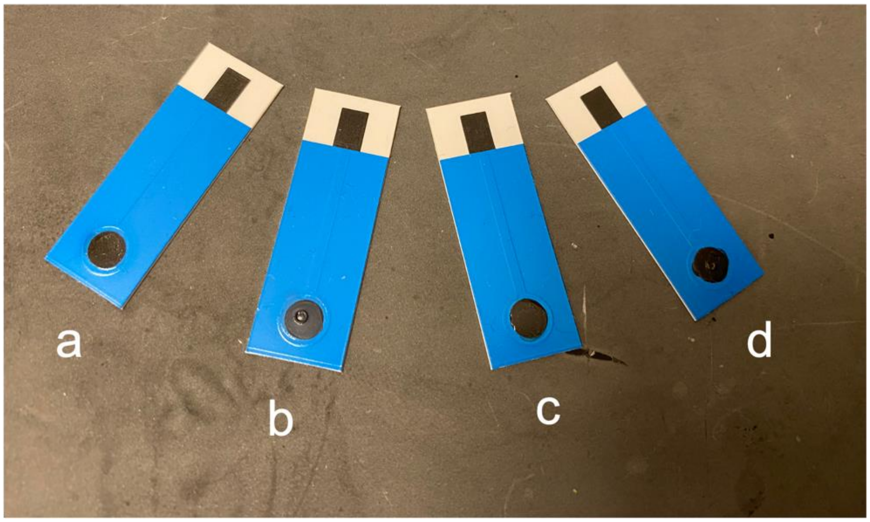

Figure S3. Photograph shows the (a) conventional Li^+ ISE sensor, (b) MXene-coated Li^+ ISE sensor, (c) MXene-PSS coated Li^+ ISE sensor and (d) MXene-LS coated Li^+ ISE sensor.

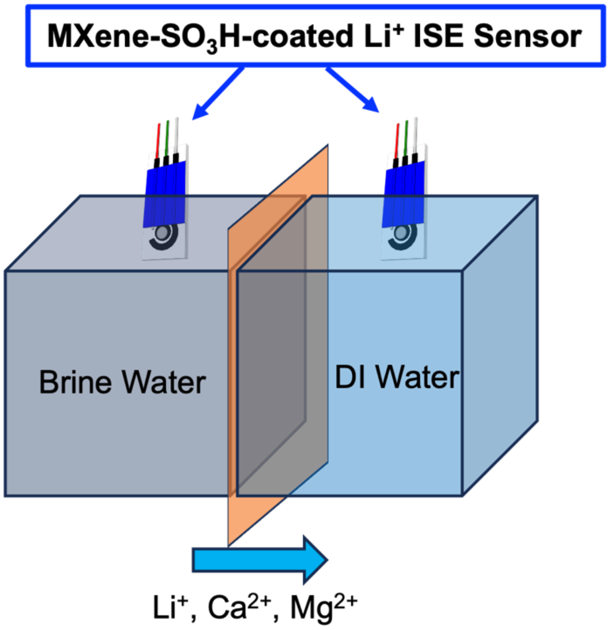

Figure S4. Schematic illustration of the experimental setup for lithium recovery.

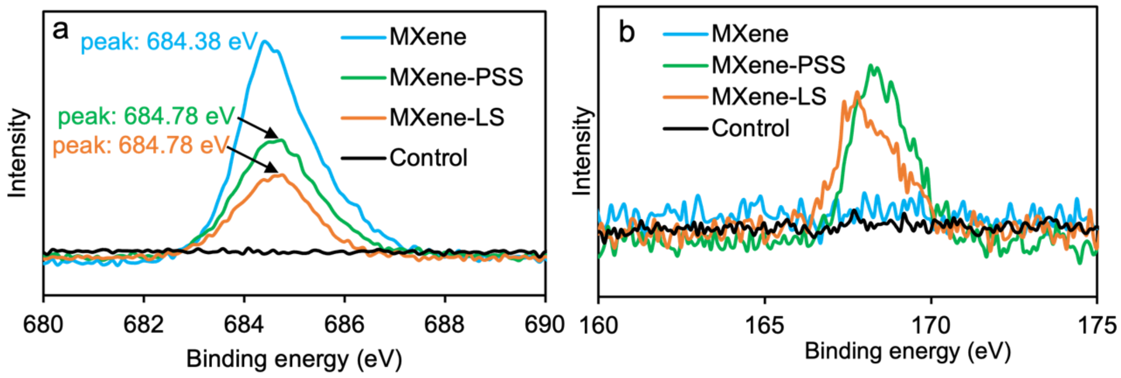

Figure S5. The XPS spectra of the conventional Li^+ ISE sensor (black), MXene-coated Li^+ ISE sensor (blue), MXene-PSS coated Li^+ ISE sensor (green) and MXene-LS coated Li^+ ISE sensor (orange). (a) The F1s scan results. (b) The S2p scan results.

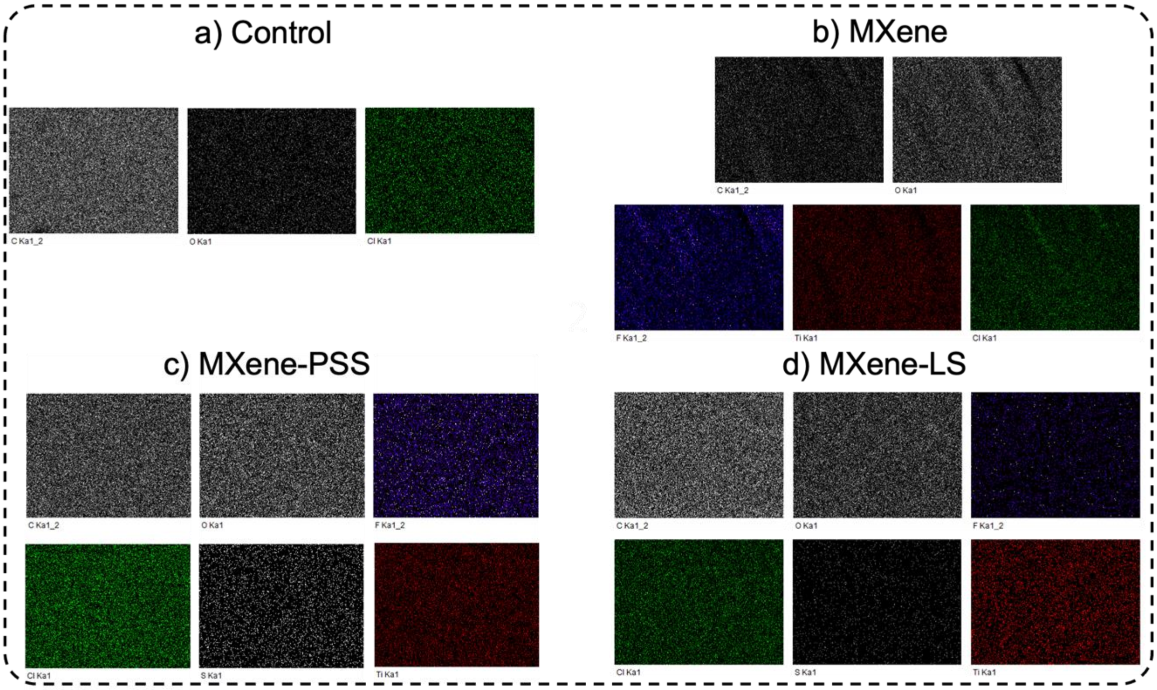

Figure S6. The EDX images of the sensor surface. (a) conventional Li^+ ISE sensor, atomic percentage (atom %): O: 54.34, Cl: 19.78, F: 0, S: 0, Ti: 0. (b) MXene-coated Li^+ ISE sensor, atom %: O: 61.86, Cl: 6.59, F: 29.29, S: 0, Ti: 10.94. (c) MXene-PSS coated Li^+ ISE sensor, atomic percentage (atom %): O: 26.47, Cl: 3.39, F: 6.09, S: 0.33, Ti: 4.40. (d) MXene-LS coated Li^+ ISE sensor. atomic percentage (atom %): O: 75.52, Cl: 10.17, F: 5.01, S: 0.21, Ti: 7.83.

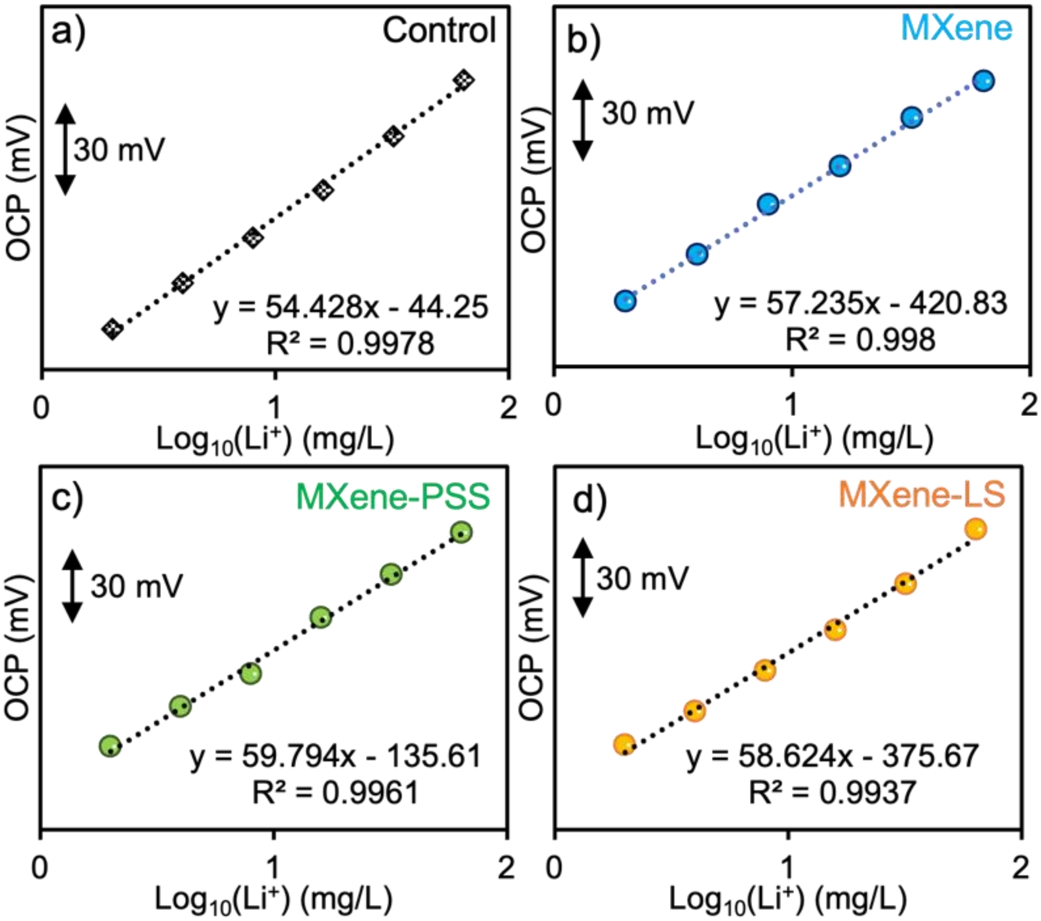

Figure S7. The calibration curves of the Li^+ ISE sensors. (a) Conventional Li^+ ISE sensor. (b) MXene-coated Li^+ ISE sensor. (c) MXene-PSS coated Li^+ ISE sensor. (d) MXene-LS coated Li^+ ISE sensor.

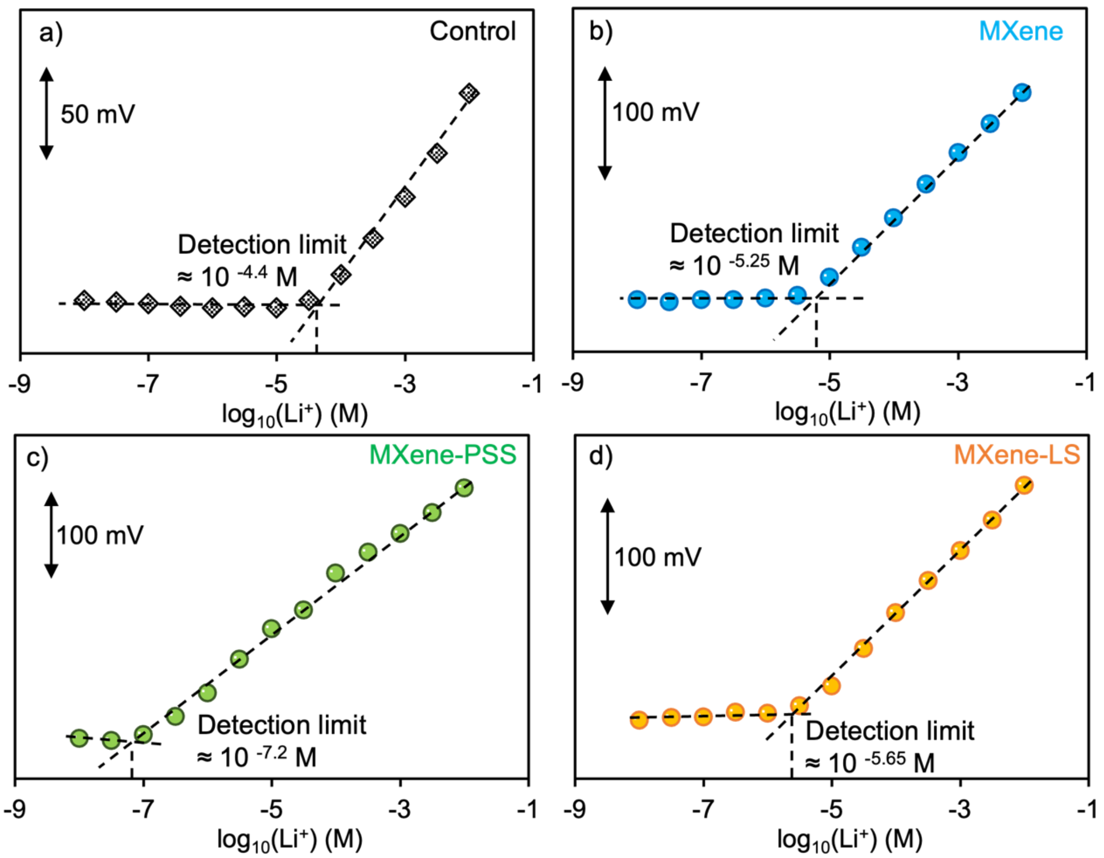

Figure S8. The detection limit (sensitivity) test of the Li^+ ISE sensors. (a) Conventional Li^+ ISE sensor. (b) MXene-coated Li^+ ISE sensor. (c) MXene-PSS coated Li^+ ISE sensor. (d) MXene-LS coated Li^+ ISE sensor.

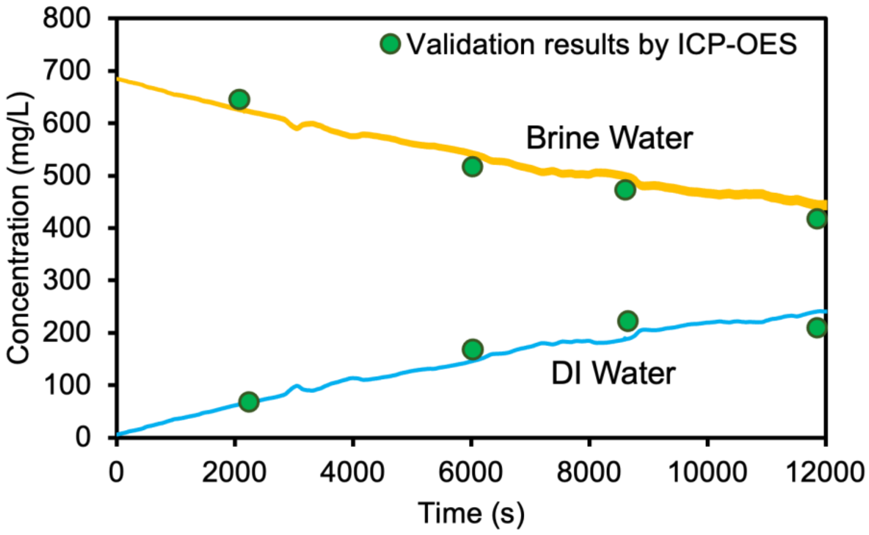

Figure S9. The MXene-PSS Li^+ ISE sensor readings under the simulated brine water recovery process. The orange line is the sensor readings on the feedwater side (simulated brine water), and the blue line is the sensor readings on the permeate side (DI water). The green dots represent the Li^+ validation results from the ICP-OES.

Table S1. The concentration of each ion in the typical water/wastewater streams for lithium recovery.

Waste Stream	Li^+ (mg/L)	Na^+ (mg/L)	K^+ (mg/L)	Ca^{2+} (mg/L)	Mg^{2+} (mg/L)	Ref.
**Brine from salt lakes**	50-1840	~100,000	~10,000	~500	~3000	[4,5]
**Wastewater from battery plants**	~1,900	~42,000	<50	200-800	200-800	[6,7]
**Geothermal water**	~25	~680	~140	~200	<10	[8,9]
**Destination plant brines**	~10	11.4-28.2	43.1-668	417-1,020	260-1,980	[10,11]

Table S2. Surface chemical compositions (atomic percentage) of the different membranes (data from XPS).

Membrane	C (%)	Cl (%)	O (%)	N (%)	Ti (%)	F (%)	S (%)
**Conventional ISE**	68.41	21.46	8.18	1.95	0	0	0
**MXene-coated ISE**	56.12	3.43	17.74	2.79	11.90	8.02	0
**MXene-PSS coated ISE**	61.24	3.44	22.37	0.28	6.04	5.25	1.38
**MXene-LS coated ISE**	64.30	6.60	19.15	2.34	3.23	3.27	0.86

Table S3. The selectivity coefficient of Li^{+} over Na^{+} ($\log k^{pot}_{Li^{+},Na^{+}}$) for different Li^{+} ISE sensors.

Concentration (mg/L)	Control	MXene	MXene-PSS	MXene-LS
0.25	-0.03	0.00	-0.07	-0.07
0.5	-0.27	-0.26	-0.40	-0.34
1	-0.62	-0.52	-0.70	-0.71
2	-0.99	-0.72	-1.02	-1.05
4	-1.30	-0.92	-1.33	-1.39
8	-1.55	-1.07	-1.58	-1.73
16	-1.76	-1.20	-1.83	-1.98
32	-1.94	-1.29	-2.04	-2.19
64	-2.13	-1.39	-2.25	-2.37
128	-2.26	-1.43	-2.37	-2.51

Table S4. The selectivity coefficient of Li^+ over K^+ ($\log k^{pot}_{Li^+,K^+}$) for different Li^+ ISE sensors.

Concentration (mg/L)	Control	MXene	MXene-PSS	MXene-LS
0.25	-0.05	-0.01	-0.10	-0.09
0.5	-0.28	-0.27	-0.39	-0.28
1	-0.61	-0.53	-0.68	-0.56
2	-0.96	-0.74	-0.97	-0.83
4	-1.30	-1.00	-1.25	-1.12
8	-1.59	-1.20	-1.51	-1.49
16	-1.84	-1.39	-1.78	-1.78
32	-2.10	-1.58	-2.05	-2.06
64	-2.28	-1.77	-2.31	-2.31
128	-2.47	-1.90	-2.54	-2.51

Table S5. The Li^+ concentration of wastewater validated using a commercialized Li^+ ISE sensor and compared with the results obtained using the conventional Li^+ ISE sensor and MXene-PSS coated Li^+ ISE sensor.

Day	Validation results (mg/L)	Conventional Li^+ ISE sensor		MXene-PSS coated Li^+ ISE sensor	
		Reading (mg/L)	Error (%)	Reading (mg/L)	Error (%)
0	10.04	10.08	0.40	10.10	0.60
1	9.87	9.87	0.00	9.26	6.18
2	10.28	8.54	16.93	10.49	2.04
3	10.15	10.65	4.93	12.19	20.10
4	10.21	11.08	8.52	11.89	16.45
5	10.45	11.64	11.39	11.70	11.96
6	10.32	17.55	70.06	11.36	10.08
7	10.15	16.98	67.29	11.35	11.82
8	10.78	16.69	54.82	10.82	0.37
9	9.98	15.25	52.81	11.31	13.33
10	10.13	15.70	54.99	10.73	5.92
11	10.27	14.93	45.37	11.45	11.49
12	10.54	15.14	43.64	11.39	8.06
13	10.79	17.30	60.33	11.59	7.41
14	10.26	17.53	70.86	12.22	19.10
Average Error			37.96		9.66

REFERENCE

(1) Han, T.; Mattinen, U.; Bobacka, J. Improving the Sensitivity of Solid-Contact Ion-Selective Electrodes by Using Coulometric Signal Transduction. *ACS Sens.* **2019**, *4* (4), 900–906.

(2) M. Zahran, E.; New, A.; Gavalas, V.; G. Bachas, L. Polymeric Plasticizer Extends the Lifetime of PVC-Membrane Ion-Selective Electrodes. *Analyst* **2014**, *139* (4), 757–763.

(3) Bakker, E.; Pretsch, E.; Bühlmann, P. Selectivity of Potentiometric Ion Sensors. *Anal. Chem.* **2000**, *72* (6), 1127–1133.

(4) Mernagh, T. P.; Bastrakov, E. N.; Jaireth, S.; de Caritat, P.; English, P. M.; Clarke, J. D. A. A Review of Australian Salt Lakes and Associated Mineral Systems. *Aust. J. Earth Sci.* **2016**, *63* (2), 131–157.

(5) Kilic, O.; Kilic, A. M. Salt Crust Mineralogy and Geochemical Evolution of the Salt Lake (Tuz Gölü), Turkey. *Sci. Res. Essays* **2010**, *5* (11), 1317–1324.

(6) Kim, S.; Kim, J.; Kim, S.; Lee, J.; Yoon, J. Electrochemical Lithium Recovery and Organic Pollutant Removal from Industrial Wastewater of a Battery Recycling Plant. *Environ. Sci. Water Res. Technol.* **2018**, *4* (2), 175–182.

(7) Gupta, S.; Babu, B. V. Removal of Toxic Metal Cr(VI) from Aqueous Solutions Using Sawdust as Adsorbent: Equilibrium, Kinetics and Regeneration Studies. *Chem. Eng. J.* **2009**, *150* (2), 352–365.

(8) Shah, M.; Sircar, A.; Varsada, R.; Vaishnani, S.; Savaliya, U.; Faldu, M.; Vaidya, D.; Bhattacharya, P. Assessment of Geothermal Water Quality for Industrial and Irrigation Purposes in the Unai Geothermal Field, Gujarat, India. *Groundw. Sustain. Dev.* **2019**, *8*, 59–68.

(9) Han, T.; Yu, X.; Guo, Y.; Li, M.; Duo, J.; Deng, T. Green Recovery of Low Concentration of Lithium from Geothermal Water by a Novel FPO/KNiFC Ion Pump Technique. *Electrochimica Acta* **2020**, *350*, 136385.

(10) Kim, S.; Joo, H.; Moon, T.; Kim, S.-H.; Yoon, J. Rapid and Selective Lithium Recovery from Desalination Brine Using an Electrochemical System. *Environ. Sci. Process. Impacts* **2019**, *21* (4), 667–676.

(11) Omerspahic, M.; Al-Jabri, H.; Siddiqui, S. A.; Saadaoui, I. Characteristics of Desalination Brine and Its Impacts on Marine Chemistry and Health, With Emphasis on the Persian/Arabian Gulf: A Review. *Front. Mar. Sci.* **2022**, *9*.
